# Hospitals

**Published:** 1879-10

**Authors:** 


### HOSPITALS.

THERE is a very general impression that hospitals are places for those who are sick and poor to obtain suitable medical aid, and there is a feeling that such persons are objects of charity, having no home and no friends. In large cities hospitals are so arranged that the very best medical appliances can be obtained, and with such conveniences, attendance, nursing, and large supply of the means for meeting accidents, that in many cases the chances of restoration to health and life are very much greater by being taken to an hospital than to a person's own home—as, for example, St. Luke's Hospital, on Fifth avenue, New York, within a short distance of the Central Park; its doors are open to all, for pay or without pay—to rich and poor alike. And, for this reason, one of the noblest of charities is to pay to the authorities three thousand dollars for the purchase of a "bed;" which means that the interest of that money will pay all the expenses of a poor person in that institution as long as the country endures. To persons who are able the charges are about twenty dollars a week.

In the early ages, when a man was sick and poor, his only hope of health was to place himself on the way side, or at the gate of a city, where, among the passing throng, there would some be found to give money, others to give advice from their own personal experience and observation.

The hospital is an outgrowth of Christian sympathy; the first ever instituted was in 660, by the good Bishop of Landsy, in Paris ; it was called The Hose of God—"Hotel Dieu." From small beginnings it reached the capacity of a thousand beds, and for centuries accommodated about nine thousand persons annually. In those early times the luxury of a bed to himself was not accorded to every one; sometimes as many as eight or ten—young and old, male and female, sick and well—were huddled together in the same bed—the sane and insane all alike. At the end of a thousand years another hospital was established in Paris, the handiwork of a lady, for males only, called "La Charité."

It was only within three hundred years that the first hospital was founded in England. In 1771 the New York Hospital was established, and it remained the only one in the whole State for seventy-five years; since when those homes for the sick have increased on every hand, and are so greatly improved that, instead of placing half a dozen in one bed, each patient has a bed to himself; instead of sending the sane and insane, males and females, sick and well to the same old, out of the way, prison-like building, the tendency is to classify, and to send those suffering from similar maladies to the same place, and to buildings which are the perfection of elegance, cleanliness, ventilation and comfort; all of which means that as the world is growing older it is growing better—more sympathetic, more humane—following the example of the Master who "went about doing good."

